# Reduced predation pressure as a potential driver of prey diversity and abundance in complex habitats

**DOI:** 10.1038/s44185-022-00007-x

**Published:** 2023-01-06

**Authors:** Chia-chen Chang, Peter A. Todd

**Affiliations:** Department of Biological Sciences, National University of Singapore, 16 Science Drive 4, Singapore, 117558 Singapore

**Keywords:** Ecology, Ecology

## Abstract

Habitat complexity is positively associated with biodiversity and abundance and is often a focus of habitat restoration programmes, however, the mechanisms underlying these relationships are not yet resolved. In this Perspective, we postulate that reduced predation pressure in complex habitats could contribute to increased prey diversity and abundance. Based on a systematic review and meta-analysis of experimental studies, reduced predation pressure in complex habitats is consistent across freshwater and marine ecosystems, field and laboratory experiments, different hunting modes of predators, and different numbers of prey species. However, the effects are less clear in terrestrial ecosystems. Easing predation pressure, in conjunction with increased resources for prey, could help explain the high biodiversity and abundance found in complex habitats. This knowledge can be used in restoration and ecological engineering projects to maximise species diversity and abundance gains.

## Habitat complexity, predation pressure and biodiversity

Even though habitat complexity can lead to increased species richness and abundance^1–3^, and is gaining traction as a strategy in restoration programmes^4–6^, exactly how complexity has these effects remains unclear. There is, however, substantial evidence that predation pressure is negatively associated with both prey diversity and biomass^7,8^ and, if predation pressure is lower in complex habitats, this could result in greater species richness and abundance. The research on the “predation pressure—complex habitat” relationship is disparate and has not yet been synthesised. Therefore, in this Perspective, we investigate whether and how the strength of predator-prey interactions differs between structurally simple versus complex habitats through a systematic review and meta-analysis of the experimental studies conducted to date.

Habitat complexity is commonly defined as the amount of, or the variation in, physical structure in space^9^. This includes density, spatial arrangement, and the number of structural types^9^. Habitat complexity is critical for both species richness and abundance^1–3^. A recent study in coral reef habitat patches showed that more than 50% of biodiversity can be explained by reef habitat structure alone^10^. The positive relationship between biodiversity/abundance and complexity could be due to greater resources, especially food and available spaces, facilitating niche differentiation and thus reducing competition and promoting coexistence^11^. Importantly, predation mediates competition, and predation may be more important than competition in regulating biodiversity^12,13^. Predation pressure can drive prey to limited predator-free habitats, which introduces competition among prey species and disturbs stable coexistence^14^. Recent ecosystem manipulations of small Caribbean islands found that the introduction of new predators destabilised the coexistence of competing prey species^14^. Predator-prey interactions have also been shown to be vital in shaping global biodiversity^15^. For example, across 291 predator removal experiments, the presence of predators consistently reduced both prey diversity and abundance^8^. As the presence of predators or increased predator richness has been found to reduce prey species diversity and abundance^7,8^, if it can be shown that complexity reduces such predation pressure, then this reduction could be an important driver underlying the positive relationship between habitat complexity and biodiversity/abundance. However, even though there exists a substantial amount of research investigating the role of habitat complexity in determining predation pressure, a clear pattern has yet to emerge^16–20^.

The inconsistent results to date may, in part, be due to differences in the hunting mode of predator species^21^. For active predators, complex habitats provide shelters where prey can hide and hinder predators’ movement when searching for prey^22^. These effects could reduce the strength of predator-prey interactions. For ambush predators, complex habitats can provide cover from where they can attack prey once it moves into their striking zone, or may offer perching sites for predators to detect prey better^23^. These effects would increase the strength of predator-prey interactions^24^. Furthermore, some predators switch from active to ambush hunting strategies when they are in simple versus complex habitats, and hence their foraging outcome might not be affected by habitat complexity to the same extent as a predator that does not have this flexibility^25,26^.

**Fig. 1:**
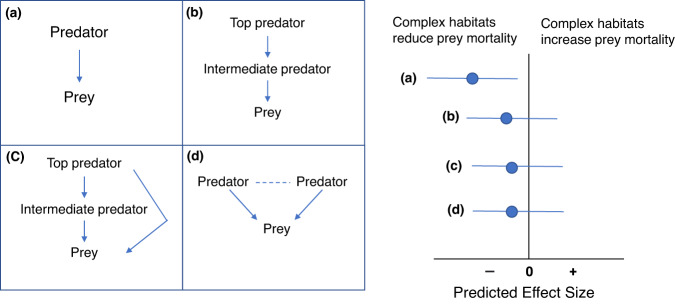
Four hypothetical scenarios and predictions. We predict that complex habitats reduce prey mortality in a simple predator-prey interaction (**a**). The presence of top predators undermines the benefits of habitat complexity for prey survival (**b**, **c**) or when there are multiple competing predator species (**d**).

The difference in predation pressure between simple and complex habitats may also be moderated by the number of trophic levels in the food web, especially in the presence of higher-level predators (i.e., top predators). The presence of top predators can reduce the density of lower-level predators (intermediate predators) or suppress their foraging activities, which benefits the survival of prey^27–29^ (Fig. 1). A previous meta-analysis^30^ of food webs that consisted of a top predator that consumed an intermediate predator, and a shared prey that was consumed by both the top and intermediate predators, found that complex habitats benefitted intermediate predators. But, because of the release of predation pressure from top predators and the resultant increased density in intermediate predators, the shared prey did not benefit from complex habitats^30^.

In addition, due to increased inter-predator competition, greater numbers of predator species have also been found to reduce the average strength of predator-prey interactions^8^. If habitat complexity weakens this competition, predation pressure could increase^31^. Under this scenario, it is possible that complex habitats would not have a clear effect on prey survival (Fig. 1). For example, consumption rates of stone crabs (*Menippe mercenaria*) and knobbed whelks (*Busycon carica*) were higher in simple habitats than in complex habitats when in isolation from the other species, but the difference was diminished when both these predator species were present^32^.

To help elucidate the effect of habitat complexity on predator-prey interactions, we conducted a meta-analysis of experimental studies. We recorded the outcomes of predator-prey interactions in simple versus complex habitats, as well as the intensity of predator foraging behaviour (e.g., the number of encounters, the number of strikes, time spent following or pursuing prey, the number of prey being detected or attacked), predator activity, prey anti-predator behaviour (e.g., the number of prey hiding, freezing, shoaling, or fleeing), and prey activity. We predicted that habitat complexity generally reduces predation pressure.

## Data collection

We performed a systematic literature search of published studies on the effect of habitat complexity on predator-prey interactions with papers published until January 14, 2021. The searching term was TS = ((“habitat complexity” OR “topological complexity” OR “structural complexity” OR “habitat heterogeneity” OR “environmental heterogeneity” OR “environmental complexity” OR “ecological complexity” OR “topographic complexity” OR “heterogeneous environments”) AND (competition OR aggress* OR prey OR predator OR predation OR forag*) AND (behav*)). We restricted our analysis to experimental studies because observational studies tended to have multiple confounding environmental or ecological factors. These experiments included at least two trophic levels where there are focal predators and focal prey (predator-prey interactions). Some experiments also contained top predators (or cues of top predators). We considered a top predator as present if there was another predator species, or cue of the predator species, that consumed the focal predators regardless of whether they also consumed the prey in the experimental setup. We compared predator-prey interactions between low(-est) vs high(-est) habitat complexity (generally topographic/structural complexity created using stone, plastic, wood, plant matter, etc.) in each experiment. Both predators and prey were always animals (e.g., herbivores were not considered as predators). To assign the level of complexity, we followed the definition within the primary study. We recorded the outcomes of predator-prey interactions (e.g., predator’s foraging success or prey’s mortality), as well as the intensity of predator’s foraging behaviour, predator’s general activity, prey’s anti-predator behaviour, and prey’s general activity. We also recorded potential moderators, i.e., the number of predator species, number of prey species, the hunting mode of predators (ambush, active, mixed, or multiple when multiple predator species with different hunting modes were used), absence/presence of the top predator, ecosystems (freshwater, marine, or terrestrial ecosystems), and field vs lab experiments. We noted the predator and prey species based on the description provided by the primary study to account for possible species-specific effects. Detailed data collection is provided in Supplementary Note 1, and lists of papers in each screening process are provided in Supplementary Data 1.

**Fig. 2:**
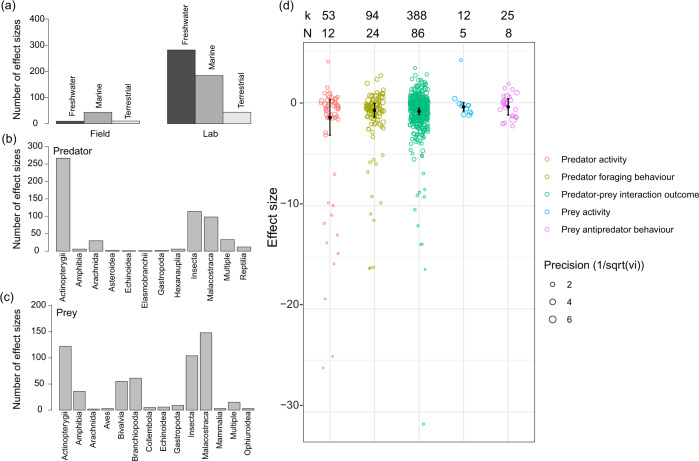
Overview of the dataset. **a** Bar plot of the number of effect sizes from each ecosystem and field vs lab experiments. **b**, **c** Bar plot of the number of effect sizes in each taxonomic group (class) for predator and prey species. “Multiple” class indicates the predator or prey species come from multiple classes. **d** Forest plot of the mean effect sizes (95% confidence interval) for predator activity, predator foraging behaviour, predator-prey interaction outcome, prey activity, and prey anti-predator behaviour. The circles indicate the raw effect sizes with the diameter of the circle indicating the precision (inverse of the square root of variance). *k* values indicate the number of effect sizes, and *N* values indicate the number of studies.

Our final dataset comprised 95 papers with 572 effect sizes (the full list is included in Supplementary Note 2). Most experiments were conducted under laboratory conditions (89%), investigating freshwater (51%) and marine (40%) systems (Fig. 2a). Animals were from 102 single predator species and 85 single prey species (21 effect sizes were from multiple predator species and 13 were from multiple prey species) across 11 and 13 taxonomic groups (Class), respectively (Fig. 2b, c). Most effects sizes were from experiments using ray-finned fish (Actinopterygii) as predators (47%; Fig. 2b) and from experiments using ray-finned fish (21%) and malacostracan crustaceans as prey (26%; Fig. 2c). We found that the sample sizes in the experiments were generally small (mean in sample sizes = 7.5, SD = 8.06), but they were mostly balanced between the simple vs complex habitats (91.8% of effect sizes having the same sample sizes). Most effect sizes (68%, 388 effect sizes from 86 papers) were from the outcome of predator-prey interactions (Fig. 2d).

## Meta-analysis

To estimate the effect of habitat complexity on predator-prey interactions, we calculated the standardised mean difference (Hedges’ g) with heteroscedastic population variances between low vs high complexity groups^33^ to establish the effect size and its sampling variance for each experiment in each study. The outcome of predator-prey interaction represents the predation pressure; higher predation success or lower prey survivorship means higher predation pressure. A positive effect size means stronger predation pressure in the complex habitat, while a negative effect size means stronger predation pressure in the simple habitat. We used multilevel meta-analytic models to estimate the overall effect of habitat complexity on predation pressure and multilevel meta-regressions to compare the effects across hunting mode of predators, number of predator species (single vs multiple), number of prey species (single vs multiple), absence/presence of the top predator, ecosystems, and field vs lab experiments. The detailed statistical analysis is provided in Supplementary Note 3. A list of hunting modes is provided in Supplementary Data 2.

## Complex habitats generally reduce predation pressure

**Fig. 3:**
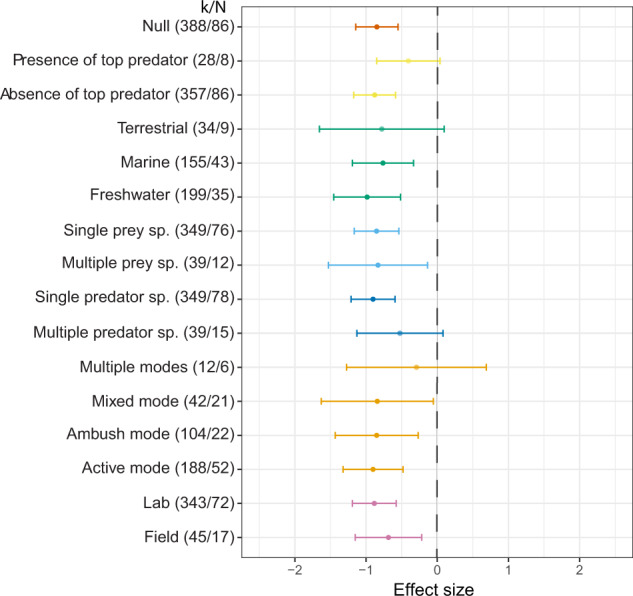
Forest plot showing the mean effect size (95% confidence intervals) of the outcome of predator-prey interactions. Negative effect sizes indicate that complex habitats reduce the predation pressure (i.e., reduced predator foraging success and improved prey survival). Values in parentheses show the number of effect size estimates (*k*) and the number of studies (*N*) within each level of moderator.

Our results indicate that complex habitats reduce predation pressure (mean effect size = −0.85, 95% CI = −1.14 to −0.55, Figs. 2d and 3); however, the effects are heterogeneous (*I*^2^ = 82%). This is mostly attributable to variation among studies (37.94%), predator species (30.93%), and prey species (13.38%). Complex habitats also reduce the intensity of predator foraging behaviour (mean effect size = −0.74, 95% CI = −1.41 to −0.07, Fig. 2d) and (non-significantly) predator activity level (mean effect size = −1.44, 95% CI = −3.16 to 0.28, Fig. 2d). Interestingly, complex habitats also reduce prey activity levels (mean effect size = −0.42, 95% CI = −0.83 to −0.01, Fig. 2d), implying that prey spend more time hiding in shelters. Heterogeneity and moderation analyses for intensity of predator foraging behaviour, predator activity, prey anti-predator behaviour, and prey activity are shown in Supplementary Tables 1 and 2. Analysis of predation pressure while controlling for predator phylogeny is provided in Supplementary Table 3 (prey phylogeny captured less than 0.1% of the variance, see Supplementary Note 3), and the result is consistent with our other analyses.

Negative effects of habitat complexity on predation pressure are consistent for both field and laboratory experiments, for experiments using either single or multiple prey species, and both marine and freshwater systems (Fig. 3). However, the results for terrestrial systems are highly variable. Additional habitat complexity studies in terrestrial systems are needed to address this deficit. Contrary to our predictions, hunting modes of predators do not influence the effect of habitat complexity on predation pressure. Unexpectedly, predation pressure is also lower in complex habitats where there are ambush predators (Fig. 3). This result is probably due to reduced prey activity in complex habitats (Fig. 2d) leading to fewer encounters with ambush predators^34^.

Predator assemblage influences the effect of habitat complexity on predation pressure. The negative effect of habitat complexity on predation pressure appears to be weaker in the presence of a top predator (presence: mean effect size = −0.41, 95% CI = −0.85 to 0.04; absence: mean effect size = −0.88, 95% CI = −1.17 to −0.59) or for experiments using multiple predator species (single predator species: mean effect size = −0.90, 95% CI = −1.21 to −0.59; multiple predator species: mean effect size = −0.52, 95% CI = −1.13 to 0.08), especially when the predators have different hunting modes (mean effect size = −0.29, 95% CI = −1.27 to 0.69).

In our dataset, we detected some small sample bias (Egger’s regression^35^ with raw effect sizes: intercept = −4.66, SE = 0.37, *P* < 0.001; Egger’s regression with residuals: intercept = −2.47, SE = 0.45, *P* < 0.001) and time-lag bias (estimate = 0.05, 95% CI = 0.02–0.08). Older papers and studies with small sample size are likely to report negative effects of habitat complexity on predation pressure. To test the robustness of the results, we re-ran the analyses excluding relatively small studies (top 10% of sampling variances) and those showing large negative effects (smallest 10% of effect sizes), nevertheless, the results are generally consistent (Supplementary Tables 4 and 5). Publication bias analyses of predator foraging behaviour, predator activity, prey activity and prey anti-predator behaviour are provided in Supplementary Table 6.

## Habitats complexity, predation pressure and habitat restoration

In this Perspective, we attempt to link two ecological phenomena that are known to influence species abundance and richness: habitat complexity^1–3^ and predation pressure^7,8^. Our results show that habitat complexity reduces predation pressure, and we posit that this is one of the mechanisms underlying the complexity–diversity relationship. Easing of predation pressure in complex habitats should mean less competition for limited predator-free space, wider foraging areas and more opportunities to use available resources—helping develop and stabilise niche differentiation and promoting prey species coexistence^14,36^. Note, our meta-analysis only examines the effects of habitat complexity on predation pressure and does not demonstrate directly an increase in species abundance and diversity.

When there were multiple predator species or in the presence of top predators, the benefits of complex habitats for the prey survival became less evident. Predation events occurring among predator species and top predators can lessen the density and foraging activity of intermediate predators, thus easing predation pressure on prey^27,28,36^. As complex habitats reduce the impact of top predators on intermediate predators, the benefits of complex habitats for prey appear to be partially cancelled out, which is consistent with a previous meta-analysis based study^30^. This result can also help explain another meta-analysis that revealed habitat complexity (more diverse plant species) had a positive effect on the abundance and diversity of natural enemies (including predators), but the abundance and diversity of pest species (the prey) were not reduced^37^, again because the increased habitat complexity, as well as increased diversity in predators, potentially cancelled each other out.

What are the implications of our findings for restoration and ecological engineering efforts that are trying to counter anthropogenic habitat simplification? Although habitat complexity plays a critical role in species richness and abundance^1,2^, identifying which habitat parameters should be manipulated continues to be a challenge^9^ because of the limited understanding of underlying mechanisms^6^. Based on data presented here, “soft” restoration efforts that create shelter from predators should lead to positive prey abundance and diversity outcomes. Similarly, ‘hard’ ecological engineering solutions, such as artificial coral reefs^38^ need to be designed in such a way as to provide appropriate refuges, for example, by matching shelter size to prey size^39^. Finally, less predation pressure may not necessarily result in the desired outcome of a restoration programme. Reducing foraging success in predators can lead to a decrease in their population size—and if the predators represent food for human consumption^40^, or are needed for pest control^41^, other strategies will likely be more appropriate.

The results of our meta-analysis show how habitat complexity leads to a reduction in predation pressure and, we argue, therefore has a positive effect on prey diversity and abundance. However, we acknowledge that we do not provide a “smoking gun” and there are undoubtedly additional mechanisms involved, such as resource availability. New research is needed, including manipulative experiments that test for the effects of complexity on biodiversity with and without predator pressure.

### Supplementary information

Supplementary Data 1

Supplementary Data 2

Supplementary Information

## Data Availability

Data and codes are available from the figshare (10.6084/m9.figshare.14844852), including the initially extracted data, list of predator and prey species and their classes (search via Kingdoms of Life Being Barcoded, http://www.barcodinglife.com/index.php/TaxBrowser_Home), data processing scripts and statistical analysis scripts.

## References

[1] Feit, B. et al. Landscape complexity promotes resilience of biological pest control to climate change. *Proc. R. Soc. B.* **288**, 20210547 (2021). 10.1098/rspb.2021.0547 PMC8150070 34034522

[2] Hall-Spencer, J. M. & Harvey, B. P. Ocean acidification impacts on coastal ecosystem services due to habitat degradation. *Emerg. Top. Life Sci.* **3**, 197–206 (2019). 33523154 10.1042/ETLS20180117 PMC7289009

[3] Loke, L. H. L. & Todd, P. A. Structural complexity and component type increase intertidal biodiversity independently of area. *Ecology* **97**, 383–393 (2016). 27145613 10.1890/15-0257.1

[4] Oliver, T. H. et al. Biodiversity and resilience of ecosystem functions. *Trends in Ecol. Evol.* **30**, 673–684 (2015). 10.1016/j.tree.2015.08.009 26437633

[5] Bullock, J. M. et al. Future restoration should enhance ecological complexity and emergent properties at multiple scales. *Ecography* ecog. **4**, 05780 (2022).

[6] Ortega, J. C. G., Thomaz, S. M. & Bini, L. M. Experiments reveal that environmental heterogeneity increases species richness, but they are rarely designed to detect the underlying mechanisms. *Oecologia* **188**, 11–22 (2018). 29736864 10.1007/s00442-018-4150-2

[7] Griffin, J. N., Byrnes, J. E. K. & Cardinale, B. J. Effects of predator richness on prey suppression: a meta-analysis. *Ecology* **94**, 2180–2187 (2013). 24358704 10.1890/13-0179.1

[8] Katano, I., Doi, H., Eriksson, B. K. & Hillebrand, H. A cross-system meta-analysis reveals coupled predation effects on prey biomass and diversity. *Oikos* **124**, 1427–1435 (2015). 10.1111/oik.02430

[9] Loke, L. H. L., Ladle, R. J., Bouma, T. J. & Todd, P. A. Creating complex habitats for restoration and reconciliation. *Ecol. Eng.* **77**, 307–313 (2015). 10.1016/j.ecoleng.2015.01.037

[10] Torres-Pulliza, D. et al. A geometric basis for surface habitat complexity and biodiversity. *Nat. Ecol. Evol.* **4**, 1495–1501 (2020). 32839543 10.1038/s41559-020-1281-8

[11] Chesson, P. Mechanisms of maintenance of species diversity. *Annu. Rev. Ecol. Syst.* **31**, 343–366 (2000). 10.1146/annurev.ecolsys.31.1.343

[12] Chesson, P. & Kuang, J. J. The interaction between predation and competition. *Nature* **456**, 235–238 (2008). 19005554 10.1038/nature07248

[13] Terborgh, J. W. Toward a trophic theory of species diversity. *Proc. Natl. Acad. Sci. USA* **112**, 11415–11422 (2015). 26374788 10.1073/pnas.1501070112 PMC4577191

[14] Pringle, R. M. et al. Predator-induced collapse of niche structure and species coexistence. *Nature* **570**, 58–64 (2019). 31168105 10.1038/s41586-019-1264-6

[15] Sandom, C. et al. Mammal predator and prey species richness are strongly linked at macroscales. *Ecology* **94**, 1112–1122 (2013). 23858651 10.1890/12-1342.1

[16] Grabowski, J. H. Habitat complexity disrupts predator-prey interactions but not the trophic cascade on oyster reefs. *Ecology* **85**, 995–1004 (2004). 10.1890/03-0067

[17] Crowder, L. B. & Cooper, W. E. Habitat structural complexity and the interaction between bluegills and their prey. *Ecology* **63**, 1802 (1982). 10.2307/1940122

[18] Almany, G. R. Does increased habitat complexity reduce predation and competition in coral reef fish assemblages? *Oikos* **106**, 275–284 (2004). 10.1111/j.0030-1299.2004.13193.x

[19] Anderson, T. L. & Semlitsch, R. D. Top predators and habitat complexity alter an intraguild predation module in pond communities. *J. Anim. Ecol.* **85**, 548–558 (2016). 26476095 10.1111/1365-2656.12462

[20] Brothers, C. A. & Blakeslee, A. M. H. Alien vs predator play hide and seek: How habitat complexity alters parasite mediated host survival. *J. Exp. Mar. Biol. Ecol.* **535**, 151488 (2021). 10.1016/j.jembe.2020.151488

[21] Horinouchi, M. et al. Seagrass habitat complexity does not always decrease foraging efficiencies of piscivorous fishes. *Mar. Ecol. Prog. Ser.* **377**, 43–49 (2009). 10.3354/meps07869

[22] Ryer, C., Stoner, A. & Titgen, R. Behavioral mechanisms underlying the refuge value of benthic habitat structure for two flatfishes with differing anti-predator strategies. *Mar. Ecol. Prog. Ser.* **268**, 231–243 (2004). 10.3354/meps268231

[23] Flynn, A. J. & Ritz, D. A. Effect of habitat complexity and predatory style on the capture success of fish feeding on aggregated prey. *J. Mar. Biol. Ass.* **79**, 487–494 (1999). 10.1017/S0025315498000617

[24] Klecka, J. & Boukal, D. S. The effect of habitat structure on prey mortality depends on predator and prey microhabitat use. *Oecologia* **176**, 183–191 (2014). 25085443 10.1007/s00442-014-3007-6

[25] James, P. L. & Heck, K. L. The effects of habitat complexity and light intensity on ambush predation within a simulated seagrass habitat. *J. Exp. Mar. Biol. Ecol.* **176**, 187–200 (1994). 10.1016/0022-0981(94)90184-8

[26] Michel, M. J. & Adams, M. M. Differential effects of structural complexity on predator foraging behavior. *Behav. Ecol.* **20**, 313–317 (2009). 10.1093/beheco/arp005

[27] Preisser, E. L., Bolnick, D. I. & Benard, M. F. Scared to death? The effects of intimidation and consumption in predator-prey interactions. *Ecology* **86**, 501–509 (2005). 10.1890/04-0719

[28] Preisser, E. L., Orrock, J. L. & Schmitz, O. J. Predator hunting mode and habitat domain alter nonconsumptive effects in predator-prey interactions. *Ecology* **88**, 2744–2751 (2007). 18051642 10.1890/07-0260.1

[29] Rypstra, A. L., Schmidt, J. M., Reif, B. D., DeVito, J. & Persons, M. H. Tradeoffs involved in site selection and foraging in a wolf spider: effects of substrate structure and predation risk. *Oikos* **116**, 853–863 (2007). 10.1111/j.0030-1299.2007.15622.x

[30] Janssen, A., Sabelis, M. W., Magalhães, S., Montserrat, M. & van der Hammen, T. Habitat structure affects intraguild predation. *Ecology* **88**, 2713–2719 (2007). 18051638 10.1890/06-1408.1

[31] Grabowski, J. H., Hughes, A. R. & Kimbro, D. L. Habitat complexity influences cascading effects of multiple predators. *Ecology* **89**, 3413–3422 (2008). 19137947 10.1890/07-1057.1

[32] Hughes, A. R. & Grabowski, J. H. Habitat context influences predator interference interactions and the strength of resource partitioning. *Oecologia* **149**, 256–264 (2006). 16705438 10.1007/s00442-006-0439-7

[33] Bonett, D. G. Meta-analytic interval estimation for standardized and unstandardized mean differences. *Psychol. Methods* **14**, 225–238 (2009). 19719359 10.1037/a0016619

[34] Huey, R. B. & Pianka, E. R. Ecological consequences of foraging mode. *Ecology* **62**, 991–999 (1981). 10.2307/1936998

[35] Egger, M., Smith, G. D., Schneider, M. & Minder, C. Bias in meta-analysis detected by a simple, graphical test. *BMJ* **315**, 629–634 (1997). 9310563 10.1136/bmj.315.7109.629 PMC2127453

[36] Ritchie, E. G. & Johnson, C. N. Predator interactions, mesopredator release and biodiversity conservation. *Ecol. Lett.* **12**, 982–998 (2009). 19614756 10.1111/j.1461-0248.2009.01347.x

[37] Chaplin-Kramer, R., O’Rourke, M. E., Blitzer, E. J. & Kremen, C. A meta-analysis of crop pest and natural enemy response to landscape complexity: pest and natural enemy response to landscape complexity. *Ecol. Lett.* **14**, 922–932 (2011). 21707902 10.1111/j.1461-0248.2011.01642.x

[38] Paxton, A. B. et al. Meta-analysis reveals artificial reefs can be effective tools for fish community enhancement but are not one-size-fits-all. *Front. Mar. Sci.* **7**, 282 (2020). 10.3389/fmars.2020.00282

[39] Eggleston, D. B., Lipcius, R. N., Miller, D. L. & Coba-Cetina, L. Shelter scaling regulates survival of juvenile Caribbean spiny lobster *Panulirus argus.**Mar. Ecol. Prog. Ser*. **62**, 79–88 (1990).

[40] Rogers, A., Blanchard, J. L. & Mumby, P. J. Fisheries productivity under progressive coral reef degradation. *J. Appl. Ecol.* **55**, 1041–1049 (2018). 10.1111/1365-2664.13051

[41] Gontijo, L. M. Engineering natural enemy shelters to enhance conservation biological control in field crops. *Biol. Control* **130**, 155–163 (2019). 10.1016/j.biocontrol.2018.10.014

