# Supplementary material for: Reduced predation pressure as a potential driver of prey diversity and abundance in complex habitats
Source: NPJ Biodivers. 2023 Jan 6;2:1. doi: 10.1038/s44185-022-00007-x (PMC11332019; doi:10.1038/s44185-022-00007-x)
Supplement: Supplementary file 3 — Supplementary Information [file 44185_2022_7_MOESM3_ESM.docx]

**Supplementary Information**

**Supplementary Note 1.**

*Literature search*

We identified relevant literature using keyword searches, including title, keywords, and abstract in Web of Science (Sci-Expanded, SSCI, A&HCI, CPCI-S, CPCI-SSH, and ESCI) and Scopus with available papers published until 14 January 2021. The searching term was TS = (("habitat complexity" OR "topological complexity" OR "structural complexity" OR "habitat heterogeneity" OR "environmental heterogeneity" OR "environmental complexity" OR "ecological complexity" OR "topographic complexity" OR "heterogeneous environments") AND (competition OR aggress* OR prey OR predator OR predation OR forag* ) AND ( behav* )). We included competition and aggression in the keyword to cover potential competition between predators for prey. We did not include thesis, books, or pre-prints. We identified 2149 articles in the initial search. The full searching strings are provided below.

Web of science:

TOPIC: (( "habitat complexity"  OR "topological complexity"  OR "structural complexity"  OR "habitat heterogeneity"  OR "environmental heterogeneity"  OR "environmental complexity"  OR "ecological complexity"  OR "topographic complexity"  OR "heterogeneous environments" )  AND (competition  OR aggress*  OR prey  OR predator  OR predation  OR forag* )  AND ( behav* )) Refined by: WEB OF SCIENCE CATEGORIES: ( ECOLOGY OR MARINE FRESHWATER BIOLOGY OR ZOOLOGY OR BEHAVIORAL SCIENCES OR EVOLUTIONARY BIOLOGY OR BIOLOGY OR FISHERIES OR OCEANOGRAPHY OR ENTOMOLOGY OR MULTIDISCIPLINARY SCIENCES OR ENVIRONMENTAL SCIENCES OR BIODIVERSITY CONSERVATION OR AGRICULTURE DAIRY ANIMAL SCIENCE OR VETERINARY SCIENCES OR LIMNOLOGY OR ORNITHOLOGY OR FORESTRY ) Timespan: All years. Indexes: SCI-EXPANDED, SSCI, A&HCI, CPCI-S, CPCI-SSH, ESCI.

Scopus:

TITLE-ABS-KEY ( "habitat complexity"  OR  "topological complexity"  OR  "structural complexity"  OR  "habitat heterogeneity"  OR  "environmental heterogeneity"  OR  "environmental complexity"  OR  "ecological complexity"  OR  "topographic complexity"  OR  "heterogeneous environments" )  AND  TITLE-ABS-KEY ( competition  OR  aggress*  OR  prey  OR  predator  OR  predation  OR  forag* )  AND  TITLE-ABS-KEY ( behav* )  AND  ( LIMIT-TO ( SUBJAREA ,  "AGRI" )  OR  LIMIT-TO ( SUBJAREA ,  "ENVI" )  OR  LIMIT-TO ( SUBJAREA ,  "MULT" ) )

After removing 656 duplicates, the remaining papers were screened by C. C. The list of papers screened and reasons for exclusion are shown in Supplementary Data 1.

Specifically, we included studies that met all the following criteria:

(1) It was an experimental study on animals. We excluded observational studies, theoretical studies, and reviews.

(2) It included measurement or manipulation of habitat complexity. Habitat complexity is defined here as the amount of information or variability in static physical components in a space ^1^. We excluded studies comparing between sites or using continuous levels of complexity in the field because these microhabitats generally had many confounded ecological variables, such as species richness, predator/prey density, or other environmental variables.

(3) Predator-prey interaction results were reported. Results included the outcome of predator-prey interactions, such as foraging success or prey mortality, and/or predator and prey behaviour that may influence the outcome of predator-prey interactions (i.e., intensity of predator’s foraging behaviour and prey’s anti-predator behaviour). We excluded studies focusing on cannibalism, choice of prey, and habitat choice between simple versus complex habitats. We also excluded studies using artificial/frozen food to simulate prey or predator cues to simulate predators. For studies that reported measurement at multiple time points (e.g., 1 day, 2 days, 3 days), the final time point was used (e.g., 3 days).

(4) At least two discrete levels of habitat complexity were tested. We excluded studies that put two habitats in one space because this experiment design involved habitat choice.

(5) The design comprised at least three replicates per experimental group.

(6) It provided mean or median, variation estimates (standard error, standard deviation, 95% confidence interval, or interquartile range), and exact sample sizes for each experimental group.

The PRISMA (Preferred Reporting Items for Systematic Reviews and Meta-Analysis ^2^)- like workflow and inclusion and exclusion checklist are shown below.

Records identified from:

Scopus 11Jan 2021

(n = 1051)

Web of Science 14 Jan 2021 (n = 1098)

Total n = 2149

Duplicate records removed (n = 656)

Records screened after removing duplicated records

(n = 1493)

Records excluded after screening abstracts

(n = 1121)

Full-text studies assessed for eligibility

(n = 372)

Full-text excluded with reasons (n = 277)

Studies included in analyses

(n = 95)

**Identification**

**Screening**

**Included**

Abstract screening checklist:

1. Is it an experimental study on animals (e.g., excluding review, simulation study, theoretical study, or observational study)? [code in Supplementary Data 1: not animal experiment, excluded 272 papers]
2. Is there any measurement or manipulation in habitat structural complexity? Structual complexity is defined as the amount of information or variability in static physical components in a space. This excludes, for example, light, turbidity, visibility, water velocity, temperature, patch size, cover/shelter size or quality, different types of substrates, background/structure color, ecological or social complexity, sensory complexity, and food distribution/availability. This also excludes comparisons made between sites, populations, or habitats with many confounded environmental variables, e.g., rural vs urban, burn vs unburn habitat, river vs lake, edge vs interior of forest, or inside vs outside of a certain boundary. [code in Supplementary Data 1: no measurement or manipulation in structural complexity, excluded 552 papers]
3. Is the result about behaviour (e.g., excluding species richness, abundance, population density, morphological or psychological changes)? [code in Supplementary Data 1: not behavioural, excluded 151 papers]
4. Did the study compare predator-prey interactions between habitats (e.g., excluding conspecific interactions, cannibalism, activity/movement pattern, or task-solving to locate food, courtship or mating behaviour, habitat choice/preference, home range, dispersal, host-parasite interaction, foraging on plants, frozen food, or baits)? [code in Supplementary Data 1: not predator-prey interaction, excluded 144 papers]
5. Abstract not available or duplicated, excluded 2 papers

Full-text screening checklist:

1. Is there any measurement or manipulation in structural complexity? [code in Supplementary Data 1: no measurement or manipulation in structural complexity, excluded 31 papers]
2. Is there predator-prey interaction in experimental results? Predator-prey interactions results include outcome of predator-prey interaction, predators’ foraging behaviour, prey anti-predator behaviour, and predator and prey activity. We excluded field experimental studies that confounded with the existing ecological variation, such as the diversity of predator/prey species or their population densities [code in Supplementary Data 1: not predator-prey interaction experiment results, excluded 57 papers]
   1. Did not use actual predator and prey animals (e.g., excluding studies using predator cue, using artificial food items or plants, or capture activity done by humans) [code in Supplementary Data 1: not actual predator and prey animal, excluded 31 papers]
   2. Predators and prey are the same species [code in Supplementary Data 1: same species, excluded 7 papers]
   3. Experiments regarded to habitat choice between simple vs complex habitats [code in Supplementary Data 1: habitat choice, excluded 43 papers], rearing environments being simple vs complex [code in Supplementary Data 1: development, excluded 15 papers], choice of prey items in simple vs complex environments [code in Supplementary Data 1: prey choice, excluded 6 papers]
   4. Rarely reported data, such as predator survival, or reaction field [excluded 3 papers]
3. Are there at least two groups differing in habitat structural complexity (e.g., excluding lack of control, complexity as continuous variable, or simple vs complex environments occurred in field environments but experiments done in the same environment in the lab)? [code in Supplementary Data 1: no control-treatment group, excluded 14 papers]
4. Are there clear mean/median, variation estimate, and sample size for each group? [code in Supplementary Data 1: lack of data, excluded 64 papers]
5. Full text not available, excluded 6 papers

This led to 95 papers from which all relevant experimental results were extracted by C. C. (2465 data points, this initial dataset is deposited at the figshare, see Data Accessibility). We extracted mean or median, variation estimates, and exact sample size for each group. For variation estimates, standard error, 95% confidence interval, and interquartile range were converted into standard deviation. Equations are included below.

$$Standard Deviation=Standard Error \times\surd Sample Size$$

$Standard Deviation=\frac{95\% Confidence interval}{3.92} \times\surd Sample Size$

$$Standard Deviation=10\%-90\% range\times\frac{80}{68}$$

$$Standard Deviation=25\%-75\% range \times\frac{50}{68}$$

When necessary, data were extracted from figures using WebPlotDigitizer ^3^. We also recorded additional information: ecosystem, field or lab experiment, manipulation of the treatments, predator and prey species, number of predator and prey species, predator and prey density, food web (to convert to absence/presence of top predators), the hunting mode of predator (if provided; literature search was done if the primary study did not provide the hunting mode), data source, and reasons for excluding any data from the analyses.

*Identification of control and treatment*

For the studies with two or more experimental groups, the one with the lower/lowest complexity was considered as the control, and the one with the higher/highest complexity was considered as the treatment. When there were multiple groups, if the lowest or the highest complexity group contained missing information or had less than three replicates, the closest alternative treatment was then used (effect sizes were unable to calculate if there were only two groups and one group with missing information). An experiment in a study may have multiple lowest or highest groups if different materials or component types were used in the manipulation; however, only a small percentage of lowest or highest groups were shared (39 shared lowest group from 11 papers and four shared highest groups from one paper among 572 effect size estimates for the final full dataset; 22 shared lowest groups from 9 papers and one shared highest groups from one paper among 388 effect size estimates for the outcome of predator-prey interactions). For experiments that crossed predator or prey density with habitat complexity, the ones with the same density were paired to estimate effect sizes.

*Categorization of effect size measures*

We categorized the effect size measures into five categories of predator-prey interaction measurements: 1) outcome of the predator-prey interaction, 2) intensity of predator’s foraging behaviour, 3) prey’s anti-predator behaviour, 4) predator’s activity, and 5) prey’s activity. The effect sizes were multiplied with a sign (1 or -1) for the interpretation purpose. Replication units were groups (i.e., multiple prey individuals with either one or multiple predator individuals).

The outcome of the predator-prey interaction includes the predator foraging success (i.e., number or percentage of prey consumed, prey killed, or prey captured, or change in abdomen width of predators), prey survival (i.e., number or percentage of prey survived, change in prey abundance, or number of prey remaining), and prey mortality or injury (i.e., number or percentage of prey died or injured, or prey loss rate). We coded the data from the predator perspective so that the positive sign indicates higher foraging success (and worse outcome for prey; predator foraging success and prey mortality have the sign “1”, and prey survival has sign “-1”).

The intensity of predator’s foraging behaviour is the behaviour that can result in prey capture, including the number of encounters between predator and prey (1), the number of prey being detected by predators (1), the number of strikes (1), number of prey being attacked (1), time spent following or pursuing prey (1), the distance between predator and prey when attacking (1), latency to attack or capture prey (-1), and time interval between strikes (-1). The sign “1” (“-1”) indicates increased (reduced) intensity in the foraging behaviour.

Prey anti-predator behaviour is the behaviour of prey as a response to the threat of a predator, including number or percentage of prey being visible or exposed (-1), fight (1), flee (1), escape (1), freeze (1), shoaled (1).

Predator’s activity and prey’s activity are the general locomotory activity level of predator and prey during the experiments, such as total time spent moving or moving speed.

*Potential moderators*

A total of six potential moderators are included to test whether these moderators influence the effects of habitat complexity on predator-prey interactions: hunting mode of predators, number of predator species, number of prey species, absence/presence of the top predator, ecosystems, and field vs lab experiments.

Predator hunting mode

Predators were categorized into three categories depending on their hunting mode: active predator, ambush predator, and mixed-mode predator. Active predators continuously search for prey ^4^. Ambush predators generally remain in a fixed location but move to pursue and capture prey (e.g., sit-and-purse or sit-and-wait)^4^. Mixed-mode predators switch between active and ambush mode depending on the environments, including habitat complexity ^5–7^.

We recorded the hunting mode from the primary study. For those that did not mention hunting mode, literature searches were conducted and, if this was unsuccessful, the hunting mode of the closest approximate (genus) was used. For studies with multiple predator species that use different hunting modes, “multiple” was coded. For studies without clear predator species, especially field experiments, “NA” was coded and removed from the analysis on this moderator. If hunting mode could not be identified from the primary study or literature search, “undetermined” was coded and removed from the analyses.

The list of predator hunting modes for each species and reference was shown in Supplementary Data 2.

Number of predator species and number of prey species

We extracted the number of predator species and prey species used in the experiment, and these moderators were coded as categorical variables (i.e., single or multiple).

Absence or presence of top predators

We coded top predators as being present when there was another predator species (or cue of top predators) that consumed the focal (i.e., intermediate) predator or consumed both focal predators and the focal prey. Specific food web scenarios used to identify either the absence or presence of top predators are included below. If experiments combined both the absence and presence of top predators, “NA” was coded and removed from this moderator analysis.

Food webs to identify absence or presence of the top predators. “Multiple” focal predators (prey) mean multiple predator (prey) species. Top predator (cue) means either actual top predator or the cue of the top predator.


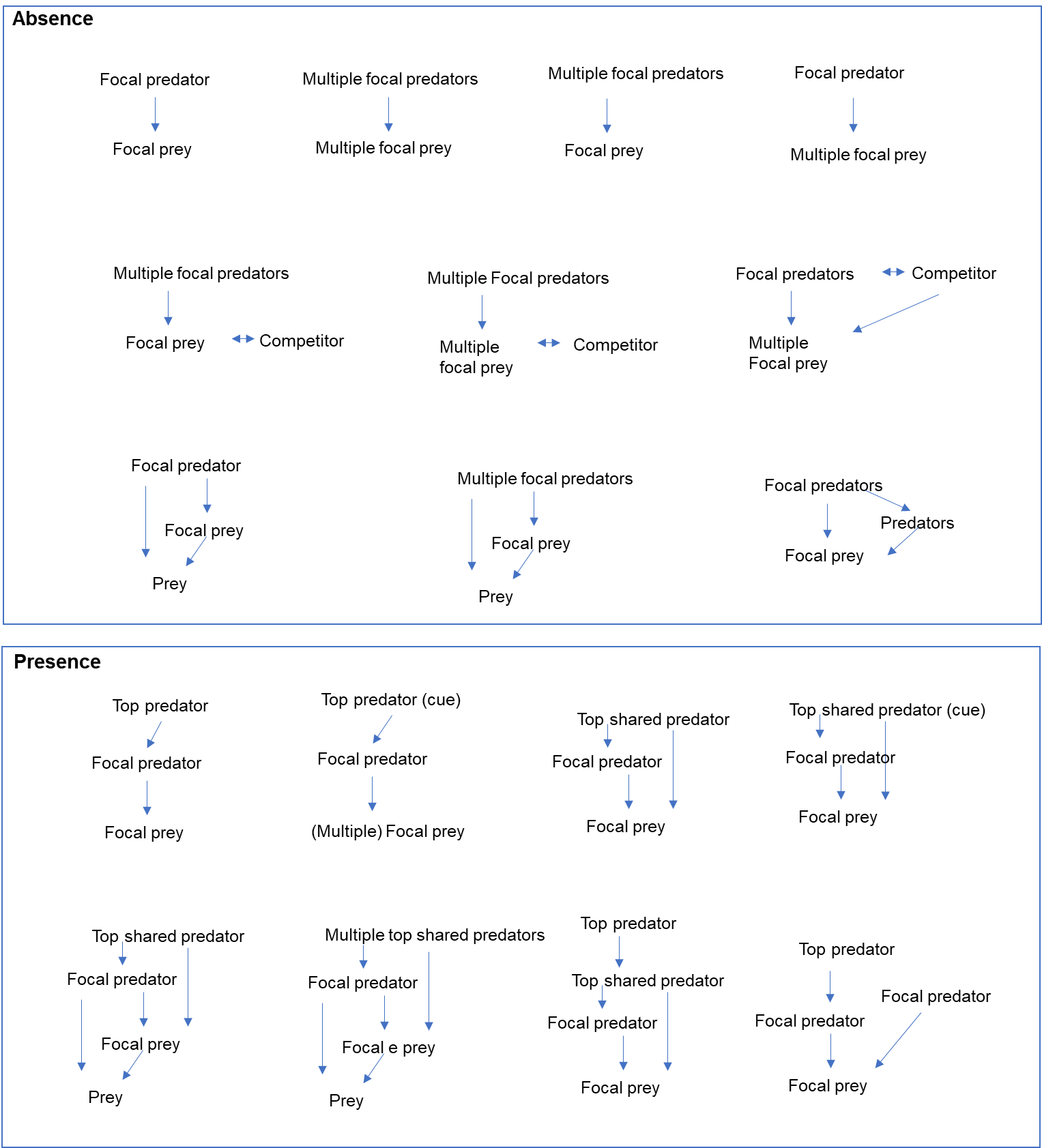


The moderator number of predator species and the absence/presence of top predators may not be independent. This is because, if a top predator species consumed both the focal predator and focal prey (intraguild predation), the number of predator species resulting in the prey mortality was coded as two (top predator species and focal predator species). In our dataset, 21% (6 out of 28) of the effect sizes with the presence of a top predator had multiple predator species that consumed focal prey, and only 8% (44 out of 541) of the effect sizes with the absence of a top predator had multiple predator species.

Ecosystems

Three ecosystems were included: marine, freshwater, and terrestrial systems. This moderator was included to explore whether the effect of habitat complexity was consistent across ecosystems.

Field vs lab experiments

Experiments conducted in outdoor tanks were considered laboratory experiments because the researchers could control environments. We include this moderator to test whether results are consistent between lab vs field experiments. It should be noted that field experiments are more likely to have multiple predator and prey species, or cues from other predators.

**Supplementary Note 2**

*List of papers used in the meta-analysis*

Adams, A. J., J. V. Locascio, and B. D. Robbins. 2004. Microhabitat use by a post-settlement stage estuarine fish: evidence from relative abundance and predation among habitats. Journal of Experimental Marine Biology and Ecology **299**:17-33.

Aguirre, J. D., and D. C. McNaught. 2013. Habitat complexity mediates predation of juvenile abalone by starfish. Marine Ecology Progress Series **487**:101-111.

Alexander, M. E., J. T. A. Dick, and N. E. O'Connor. 2013a. Born to kill: Predatory functional responses of the littoral amphipod Echinogammarus marinus Leach throughout its life history. Journal of Experimental Marine Biology and Ecology **439**:92-99.

Alexander, M. E., J. T. A. Dick, and N. E. O'Connor. 2013b. Trait-mediated indirect interactions in a marine intertidal system as quantified by functional responses. Oikos **122**:1521-1531.

Almany, G. R. 2004a. Differential effects of habitat complexity, predators and competitors on abundance of juvenile and adult coral reef fishes. Oecologia **141**:105-113.

Almany, G. R. 2004b. Does increased habitat complexity reduce predation and competition in coral reef fish assemblages? Oikos **106**:275-284.

Amaral, D. S. S. L., M. Venzon, A. L. Perez, J. M. Schmidt, and J. D. Harwood. 2015. Coccinellid interactions mediated by vegetation heterogeneity. Entomologia Experimentalis Et Applicata **156**:160-169.

Anderson, T. L., and R. D. Semlitsch. 2016. Top predators and habitat complexity alter an intraguild predation module in pond communities. Journal of Animal Ecology **85**:548-558.

Anderson, T. W. 2001. Predator responses, prey refuges, and density-dependent mortality of a marine fish. Ecology **82**:245-257.

Bartholomew, A. 2002. Total cover and cover quality: predicted and actual effects on a predator's foraging success. Marine Ecology Progress Series **227**:1-9.

Brothers, C. A., and A. M. H. Blakeslee. 2021. Alien vs predator play hide and seek: How habitat complexity alters parasite mediated host survival. Journal of Experimental Marine Biology and Ecology **535**.

Burks, R. L., E. Jeppesen, and D. M. Lodge. 2001. Littoral zone structures as Daphnia refugia against fish predators. Limnology and Oceanography **46**:230-237.

Byers, J. E., Z. C. Holmes, and J. C. Malek. 2017. Contrasting complexity of adjacent habitats influences the strength of cascading predatory effects. Oecologia **185**:107-117.

Carr, L. A., and K. E. Boyer. 2014. Variation at multiple trophic levels mediates a novel seagrass-grazer interaction. Marine Ecology Progress Series **508**:117-128.

Carroll, J. M., L. J. Jackson, and B. J. Peterson. 2015. The Effect of Increasing Habitat Complexity on Bay Scallop Survival in the Presence of Different Decapod Crustacean Predators. Estuaries and Coasts **38**:1569-1579.

Chandler, H. C., T. A. Gorman, and C. A. Haas. 2016. The Effects of Crayfish Predation and Vegetation Cover on Tadpole Growth, Survival, and Nonlethal Injury. Journal of Herpetology **50**:271-277.

Corkum, L. D., and D. J. Cronin. 2004. Habitat complexity reduces aggression and enhances consumption in crayfish. Journal of Ethology **22**:23-27.

Corona, A., L. A. Soto, and A. J. Sanchez. 2000. Epibenthic amphipod abundance and predation efficiency of the pink shrimp Farfantepenaeus duorarum (Burkenroad, 1939) in habitats with different physical complexity in a tropical estuarine system. Journal of Experimental Marine Biology and Ecology **253**:33-48.

Cuthbert, R. N., J. T. A. Dick, and A. Callaghan. 2018. Interspecific variation, habitat complexity and ovipositional responses modulate the efficacy of cyclopoid copepods in disease vector control. Biological Control **121**:80-87.

Czarnecka, M., T. Kakareko, L. Jermacz, R. Pawlak, and J. Kobak. 2019. Combined effects of nocturnal exposure to artificial light and habitat complexity on fish foraging. Science of the Total Environment **684**:14-22.

Davis, M. J., J. L. Purrenhage, and M. D. Boone. 2012. Elucidating Predator-Prey Interactions Using Aquatic Microcosms: Complex Effects of a Crayfish Predator, Vegetation, and Atrazine on Tadpole Survival and Behavior. Journal of Herpetology **46**:527-534.

DeBoom, C. S., and D. H. Wahl. 2013. Effects of Coarse Woody Habitat Complexity on Predator-Prey Interactions of Four Freshwater Fish Species. Transactions of the American Fisheries Society **142**:1602-1614.

Drolet, D., J. H. Himmelman, and R. Rochette. 2004. Use of refuges by the ophiuroid Ophiopholis aculeata: contrasting effects of substratum complexity on predation risk from two predators. Marine Ecology Progress Series **284**:173-183.

Dunn, R. P., A. H. Altieri, K. Miller, M. E. Yeager, and K. A. Hovel. 2017. Coral identity and structural complexity drive habitat associations and demographic processes for an increasingly important Caribbean herbivore. Marine Ecology Progress Series **577**:33-47.

Ferner, M. C., D. L. Smee, and M. J. Weissburg. 2009. Habitat complexity alters lethal and non-lethal olfactory interactions between predators and prey. Marine Ecology Progress Series **374**:13-22.

Figueiredo, B. R., R. P. Mormul, and E. Benedito. 2014. Structural complexity and turbidity do not interact to influence predation rate and prey selectivity by a small visually feeding fish. Marine and Freshwater Research **66**:170-176.

Figueiredo, B. R. S., L. F. Fiori, F. W. Keppeler, R. P. Mormul, and E. Benedito. 2018. Non-lethal effects of a native and a non-native piscivorous fish on the interaction between a mesopredator and benthic and pelagic invertebrates. Aquatic Invasions **13**:553-563.

Finke, D. L., and R. F. Denno. 2006. Spatial refuge from intraguild predation: implications for prey suppression and trophic cascades. Oecologia **149**:265-275.

Glaspie, C. N., and R. D. Seitz. 2018. Habitat complexity and benthic predator-prey interactions in Chesapeake Bay. PloS one **13**.

Gontijo, L. M., D. C. Margolies, J. R. Nechols, and R. A. Cloyd. 2010. Plant architecture, prey distribution and predator release strategy interact to affect foraging efficiency of the predatory mite Phytoseiulus persimilis (Acari: Phytoseiidae) on cucumber. Biological Control **53**:136-141.

Grabowski, J. H. 2004. Habitat complexity disrupts predator–prey interactions but not the trophic cascade on oyster reefs. Ecology **85**:995-1004.

Grabowski, J. H., A. R. Hughes, and D. L. Kimbro. 2008. Habitat Complexity Influences Cascading Effects of Multiple Predators. Ecology **89**:3413-3422.

Grabowski, J. H., and S. P. Powers. 2004. Habitat complexity mitigates trophic transfer on oyster reefs. Marine Ecology Progress Series **277**:291-295.

Granzotti, R. V., C. M. Muniz, and L. C. Gomes. 2018. Habitat complexity does not influence prey consumption in an experimental three-level trophic chain. Iheringia. Série Zoologia **108**.

Grutters, B. M. C., B. J. A. Pollux, W. C. E. P. Verberk, and E. S. Bakker. 2015. Native and Non-Native Plants Provide Similar Refuge to Invertebrate Prey, but Less than Artificial Plants. PloS one **10**.

Hauzy, C., T. Tully, T. Spataro, G. Paul, and R. Arditi. 2010. Spatial heterogeneity and functional response: an experiment in microcosms with varying obstacle densities. Oecologia **163**:625-636.

Holsman, K. K., P. S. McDonald, P. A. Barreyro, and D. A. Armstrong. 2010. Restoration through eradication? Removal of an invasive bioengineer restores some habitat function for a native predator. Ecological Applications **20**:2249-2262.

Horinouchi, M., N. Mizuno, Y. Jo, M. Fujita, M. Sano, and Y. Suzuki. 2009. Seagrass habitat complexity does not always decrease foraging efficiencies of piscivorous fishes. Marine Ecology Progress Series **377**:43-49.

Hovel, K. A., A. M. Warneke, S. P. Virtue-Hilborn, and A. E. Sanchez. 2016. Mesopredator foraging success in eelgrass (Zostera marina L.): Relative effects of epiphytes, shoot density, and prey abundance. Journal of Experimental Marine Biology and Ecology **474**:142-147.

Humphries, A. T., M. K. La Peyre, and G. A. Decossas. 2011. The Effect of Structural Complexity, Prey Density, and "Predator-Free Space" on Prey Survivorship at Created Oyster Reef Mesocosms. PloS one **6**.

Isaksson, I., L. Pihl, and J. Vanmontfrans. 1994. Eutrophication-Related Changes in Macrovegetation and Foraging of Young Cod (Gadus-Morhua L) - a Mesocosm Experiment. Journal of Experimental Marine Biology and Ecology **177**:203-217.

James, P. L., and K. L. Heck. 1994. The Effects of Habitat Complexity and Light-Intensity on Ambush Predation within a Simulated Seagrass Habitat. Journal of Experimental Marine Biology and Ecology **176**:187-200.

Johnson, D. W. 2006. Predation, habitat complexity, and variation in density-dependent mortality of temperate reef fishes. Ecology **87**:1179-1188.

Jordan, F. 2002. Field and laboratory evaluation of habitat use by rainwater killifish (Lucania parva) in the St. Johns River estuary, Florida. Estuaries **25**:288-295.

Katayama, M. 2014. Stem stiffness plays a role in determining the foraging success of predators. Hydrobiologia **732**:173-181.

Keiser, C. N., S. J. Ingley, B. J. Toscano, I. Scharf, and J. N. Pruitt. 2018. Habitat complexity dampens selection on prey activity level. Ethology **124**:25-32.

Kenyon, R. A., N. R. Loneragan, and J. M. Hughes. 1995. Habitat Type and Light Affect Sheltering Behavior of Juvenile Tiger Prawns (Penaeus-Esculentus Haswell) and Success Rates of Their Fish Predators. Journal of Experimental Marine Biology and Ecology **192**:87-105.

Klecka, J., and D. S. Boukal. 2014. The effect of habitat structure on prey mortality depends on predator and prey microhabitat use. Oecologia **176**:183-191.

Kolar, V., D. S. Boukal, and A. Sentis. 2019. Predation risk and habitat complexity modify intermediate predator feeding rates and energetic efficiencies in a tri-trophic system. Freshwater Biology **64**:1480-1491.

Larranaga, N., and S. O. Steingrimsson. 2015. Shelter availability alters diel activity and space use in a stream fish. Behavioral Ecology **26**:578-586.

Ledbetter, J. F., and K. A. Hovel. 2020. Effects of a habitat-modifying eelgrass epibiont on predator success and epifaunal survival. Journal of Experimental Marine Biology and Ecology **526**.

Legrand, A., and P. Barbosa. 2003. Plant morphological complexity impacts foraging efficiency of adult Coccinella septempunctata L. (Coleoptera : Coccinellidae). Environmental Entomology **32**:1219-1226.

Macia, A., K. G. S. Abrantes, and J. Paula. 2003. Thorn fish Terapon jarbua (Forskal) predation on juvenile white shrimp Penaeus indicus H. Milne Edwards and brown shrimp Metapenaeus monoceros (Fabricius): the effect of turbidity, prey density, substrate type and pneumatophore density. Journal of Experimental Marine Biology and Ecology **291**:29-56.

Manatunge, J., T. Asaeda, and T. Priyadarshana. 2000. The influence of structural complexity on fish-zooplankton interactions: A study using artificial submerged macrophytes. Environmental Biology of Fishes **58**:425-438.

Mattila, J. 1992. The Effect of Habitat Complexity on Predation Efficiency of Perch Perca-Fluviatilis L and Ruffe Gymnocephalus-Cernuus (L). Journal of Experimental Marine Biology and Ecology **157**:55-67.

Mayer, C. M., L. G. Rudstam, E. L. Mills, S. G. Cardiff, and C. A. Bloom. 2001. Zebra mussels (Dreissena polymorpha), habitat alteration, and yellow perch (Perca flavescens) foraging: system-wide effects and behavioural mechanisms. Canadian Journal of Fisheries and Aquatic Sciences **58**:2459-2467.

Menezes, L. C. C. R., M. N. Rossi, and W. A. C. Godoy. 2006. The effect of refuge on Dermestes ater (Coleoptera : Dermestidae) predation on Musca domestica (Diptera : Muscidae): Refuge for prey or the predator? Journal of Insect Behavior **19**:717-729.

Michel, M. J. 2012. Phenotypic plasticity in complex environments: effects of structural complexity on predator- and competitor-induced phenotypes of tadpoles of the wood frog, Rana sylvatica. Biological Journal of the Linnean Society **105**:853-863.

Michel, M. J., and M. M. Adams. 2009. Differential effects of structural complexity on predator foraging behavior. Behavioral Ecology **20**:313-317.

Mislan, K. A. S., and R. C. Babcock. 2008. Survival and behaviour of juvenile red rock lobster, Jasus edwardsii, on rocky reefs with varying predation pressure and habitat complexity. Marine and Freshwater Research **59**:246-253.

Miyashita, L. K., J. P. Richardson, and J. E. Duffy. 2016. Effects of predator richness and habitat heterogeneity on prey suppression in an estuarine food chain. Marine Ecology Progress Series **559**:13-20.

Morice, S., S. Pincebourde, F. Darboux, W. Kaiser, and J. Casas. 2013. Predator-Prey Pursuit-Evasion Games in Structurally Complex Environments. Integrative and Comparative Biology **53**:767-779.

Mullin, S. J., and R. J. Cooper. 2000. The foraging ecology of the gray rat snake (Elaphe obsoleta spiloides). II. Influence of habitat structural complexity when searching for arboreal avian prey. Amphibia-Reptilia **21**:211-222.

Mullin, S. J., R. J. Cooper, and W. H. N. Gutzke. 1998. The foraging ecology of the gray rat snake (Elaphe obsoleta spiloides). III. Searching for different prey types in structurally varied habitats. Canadian Journal of Zoology **76**:548-555.

Mullin, S. J., and W. H. N. Gutzke. 1999. The foraging ecology of the gray rat snake (Elaphe obsoleta spiloides). I. Influence of habitat structural complexity when searching for mammalian prey. Herpetologica **55**:18-28.

Nelson, W. G., and E. Bonsdorff. 1990. Fish Predation and Habitat Complexity - Are Complexity Thresholds Real. Journal of Experimental Marine Biology and Ecology **141**:183-194.

Nordstrom, M., and D. M. Booth. 2007. Drift algae reduce foraging efficiency of juvenile flatfish. Journal of Sea Research **58**:335-341.

Nystrom, P., and J. R. Perez. 1998. Crayfish predation on the common pond snail (Lymnaea stagnalis): the effect of habitat complexity and snail size on foraging efficiency. Hydrobiologia **368**:201-208.

Persson, L., and P. Eklov. 1995. Prey Refuges Affecting Interactions between Piscivorous Perch and Juvenile Perch and Roach. Ecology **76**:70-81.

Petren, K., and T. J. Case. 1998. Habitat structure determines competition intensity and invasion success in gecko lizards. Proceedings of the National Academy of Sciences of the United States of America **95**:11739-11744.

Pirtle, J. L., G. L. Eckert, and A. W. Stoner. 2012. Habitat structure influences the survival and predator-prey interactions of early juvenile red king crab Paralithodes camtschaticus. Marine Ecology Progress Series **465**:169-184.

Priyadarshana, T., and T. Asaeda. 2007. Swimming restricted foraging behavior of two zooplanktivorous fishes Pseudorasbora parva and Rasbora daniconius (Cyprinidae) in a simulated structured environment. Environmental Biology of Fishes **80**:473-486.

Ray-Culp, M., M. Davis, and A. W. Stoner. 1999. Predation by xanthid crabs on early post-settlement gastropods: the role of prey size, prey density, and habitat complexity. Journal of Experimental Marine Biology and Ecology **240**:303-321.

Ren, Y., D. Xie, B. Li, Y. Liu, S. Hu, H. Liu, Y. Shi, and S. Zhu. 2020. Influence of water temperature, habitat complexity and light on the predatory performance of the dark sleeper Odontobutis potamophila (Günther, 1861). Journal of Freshwater Ecology **35**:367-378.

Ren, Y., M. T. Xiong, J. X. Yu, W. Li, B. Li, J. S. Liu, and T. L. Zhang. 2019. Effects of artificial submersed vegetation on consumption and growth of mandarin fish Siniperca chuatsi (Basilewsky) foraging on live prey. Journal of Freshwater Ecology **34**:433-444.

Renick, V. C., T. W. Anderson, S. G. Morgan, and G. N. Cherr. 2015. Interactive effects of pesticide exposure and habitat structure on behavior and predation of a marine larval fish. Ecotoxicology **24**:391-400.

Reynolds, P. G., and K. Cuddington. 2012. Effects of Plant Gross Morphology on Predator Consumption Rates. Environmental Entomology **41**:508-515.

Richardson, J. M. L., M. S. Gunzburger, and J. Travis. 2006. Variation in predation pressure as a mechanism underlying differences in numerical abundance between populations of the poeciliid fish Heterandria formosa. Oecologia **147**:596-605.

Ryer, C. H., A. W. Stoner, and R. H. Titgen. 2004. Behavioral mechanisms underlying the refuge value of benthic habitat structure for two flatfishes with differing anti-predator strategies. Marine Ecology Progress Series **268**:231-243.

Rypstra, A. L., J. M. Schmidt, B. D. Reif, J. DeVito, and M. H. Persons. 2007. Tradeoffs involved in site selection and foraging in a wolf spider: effects of substrate structure and predation risk. Oikos **116**:853-863.

Santos, A. F. G. N., E. Garcia-Berthou, C. Hayashi, and L. N. Santos. 2013. When habitat complexity increases predation risk: experiments with invasive and neotropical native fishes. Marine and Freshwater Research **64**:752-760.

Santos, A. F. G. N., L. N. Santos, E. Garcia-Berthou, and C. Hayashi. 2009. Could native predators help to control invasive fishes? Microcosm experiments with the Neotropical characid, Brycon orbignyanus. Ecology of Freshwater Fish **18**:491-499.

Savino, J. F., and R. A. Stein. 1982. Predator-Prey Interaction between Largemouth Bass and Bluegills as Influenced by Simulated, Submersed Vegetation. Transactions of the American Fisheries Society **111**:255-266.

Savino, J. F., and R. A. Stein. 1989. Behavioural interactions between fish predators and their prey: effects of plant density. Animal behaviour **37**:311-321.

Shoji, J., K. Sakiyama, M. Hori, G. Yoshida, and M. Hamaguchi. 2007. Seagrass habitat reduces vulnerability of red sea bream Pagrus major juveniles to piscivorous fish predator. Fisheries Science **73**:1281-1285.

Shoup, D. E., and C. R. Broderius. 2018. Effects of Vegetation Density on the Ontogeny to Piscivory of Juvenile Largemouth Bass. North American Journal of Fisheries Management **38**:630-638.

Siddon, C. E., and J. D. Witman. 2004. Behavioral indirect interactions: Multiple predator effects and prey switching in the rocky subtidal. Ecology **85**:2938-2945.

Srivastava, D. S. 2006. Habitat structure, trophic structure and ecosystem function: interactive effects in a bromeliad-insect community. Oecologia **149**:493-504.

Stewart, T. W., J. C. Gafford, J. G. Miner, and R. L. Lowe. 1999. Dreissena-shell habitat and antipredator behavior: combined effects on survivorship of snails co-occurring with molluscivorous fish. Journal of the North American Benthological Society **18**:274-283.

Stoner, A. W. 2009. Habitat-mediated survival of newly settled red king crab in the presence of a predatory fish: Role of habitat complexity and heterogeneity. Journal of Experimental Marine Biology and Ecology **382**:54-60.

Stuart-Smith, R. D., J. F. Stuart-Smith, R. W. G. White, and L. A. Barmuta. 2007. The impact of an introduced predator on a threatened galaxiid fish is reduced by the availability of complex habitats. Freshwater Biology **52**:1555-1563.

Stunz, G. W., and T. J. Minello. 2001. Habitat-related predation on juvenile wild-caught and hatchery-reared red drum Sciaenops ocellatus (Linnaeus). Journal of Experimental Marine Biology and Ecology **260**:13-25.

Thompson, K. A., J. E. Hill, and L. G. Nico. 2012. Eastern mosquitofish resists invasion by nonindigenous poeciliids through agonistic behaviors. Biological Invasions **14**:1515-1529.

Yarnall, A. H., and F. J. Fodrie. 2020. Predation patterns across states of landscape fragmentation can shift with seasonal transitions. Oecologia **193**:403-413.

Yeager, M. E., and K. A. Hovel. 2017. Structural complexity and fish body size interactively affect habitat optimality. Oecologia **185**:257-267.

**Supplementary Note 3.**

*Data analysis*

The meta-analysis was performed in R version 4.0.2 ^8^ using the metafor package version 2.4.0 ^9^. We estimated the standardized mean difference (Hedges’ g) with heteroscedastic population variances in two groups ^10^ for each effect size and their sampling variance using the escalc function (measure = SMDH). Some effect sizes could not be calculated, for example, when SD for both control and treatment were zero. These data were excluded from the analysis.

To estimate the overall mean effect size, we ran multilevel random-effect models (using the rma.mv function with restricted maximum likelihood) for each category of effect size measures. Predator species, prey species, and study ID were included as random effects to account for non-independence. Predator and prey species were included to estimate the amount of variance attributed to species. For studies with multiple species, all the species names were included (e.g., “*Daphnia magna* and *Simocephalus* sp.”). For studies without species name, the information provided in the literature was then used (e.g., “Chironomidae larvae”). Study ID was included because each study may contribute multiple effect sizes, such as different species, or varying density or body size. The heterogeneity of effect size was estimated using I^2^ (the amount of heterogeneity relative to the total amount of variance) and calculated for each level of random effects ^11^. To estimate the effect size for each moderator, we ran multilevel mixed-effects models, which we included a fixed effect (each moderator was run in separate models) but excluding intercept (-1) with restricted maximum likelihood. We only included a fixed effect in a model because moderators are likely to be confounded (e.g., field experiments are more likely to have multiple predator/prey species). If the 95% confidence interval of an effect size overlapped with zero, it was considered not statistically significant, and the effect of habitat complexity unclear.

We performed another set of analyses controlling for phylogeny. When controlling for predator phylogeny (327 effect sizes), 27.94% heterogeneity is attributed to predator species, 11.05% to prey species, 42.5% to different studies, 3.56% to predator phylogeny. When controlling for both predator and prey phylogeny (260 effect sizes), 30.41% heterogeneity is attributed to predator species, 12.47% to prey species, 39.07% to different studies, 4.26% to predator phylogeny, and less than 0.1% to prey phylogeny. For some studies involving multiple species or without a species name (especially for field studies), those effect sizes were not used in this set of analyses. To inccorporate phylogeny, we extracted information from the Open Tree of Life (OpenTree) database using the rotl package version 3.0.11 ^12^. We computed branch lengths using the Grafen method with height set to 1 from the ape packages version 5.4.1 ^13^, and the phylogenetic variance-covariance matrix was then added into the multilevel random-effect model or mixed-effects models mentioned above. The predator and prey phylogenetic trees are included in Supplementary Figure 1 and Supplementary Figure 2.

**
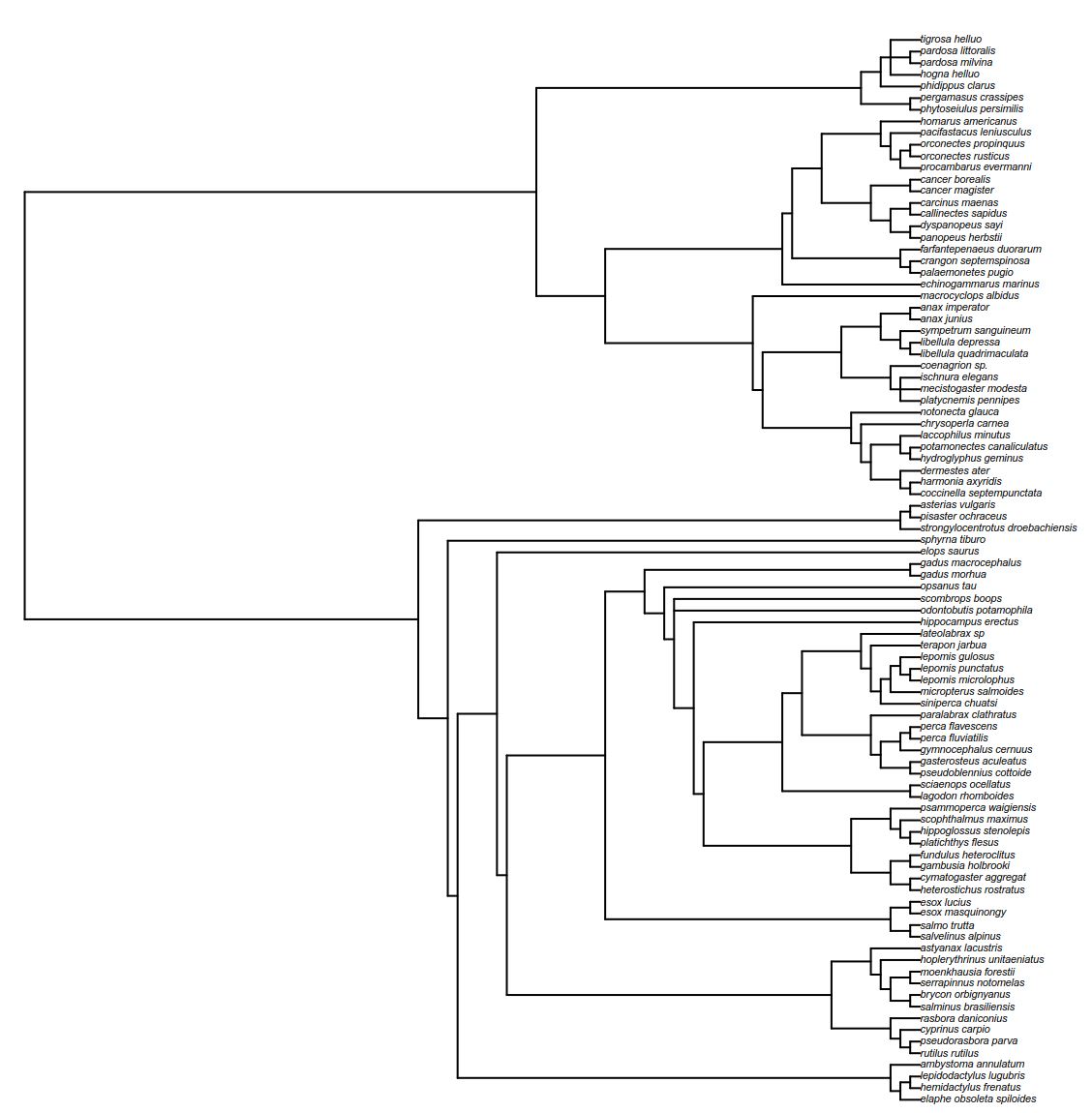
**

Supplementary Figure 1. Phylogenetic tree of predator species


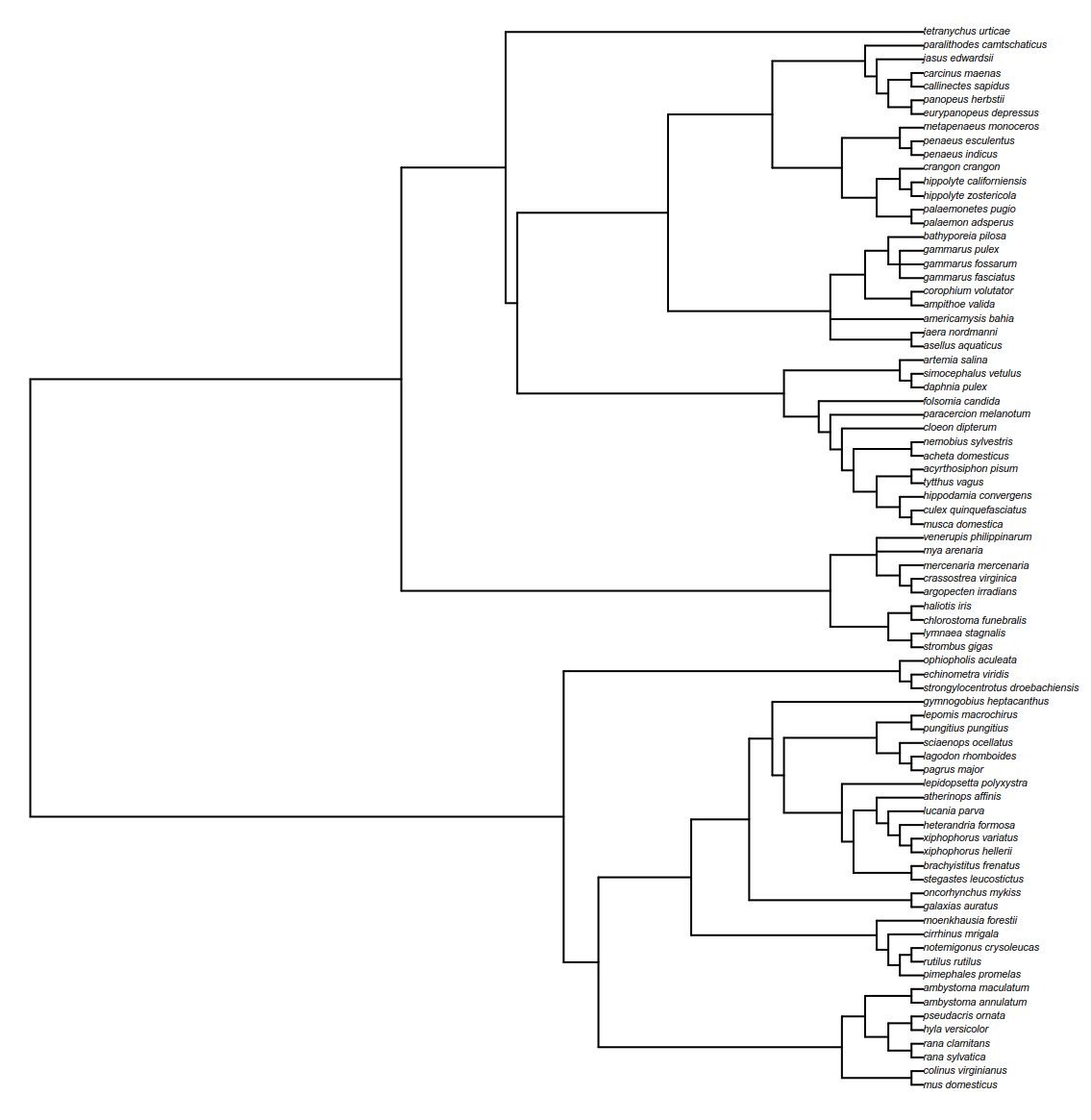
 Supplementary Figure 2. Phylogenetic tree of prey species

We performed several tests to check publication bias. First, we visually inspected traditional funnel plots ^14^. The funnel plot is based on the assumption that studies with high precision (inverse standard error; large sample sizes) tend to place near the average effect size and studies with low precision tend to spread across both sides of the average. The asymmetry of the funnel shape was then tested statistically by running Egger’s regressions (i.e., linear regressions of effect sizes on inverse standard error). However, the traditional funnel plot assumes no heterogeneity; therefore, we also plotted residual funnel plots using the meta-analytic residual (using the rma.mv function with all the moderators and random effects) and again ran Egger’s regressions ^11^.

We also tested p-hacking by visually inspecting p-curves ^15^. P-hacking is suggested when studies reporting p-value close to 0.05 are over-represented. The p-curve was performed using source code from ^16^.

Lastly, we tested time-lag bias in the effect size ^11^ by running multilevel mixed-effect models with the year of publication as a fixed effect (using the rma.mv function), and the random effects were predator species, prey species, and study ID. We ran this because, during the development of a research field, the effect sizes tend to become smaller over time ^17^.

The publication bias plots are shown in Supplementary Figure 3.

We detected some small study bias and time-lag bias. To test its influence on the conclusion, we removed relatively small studies and studies with large negative effect sizes (i.e., excluding studies within the top 10% of sampling variances or bottom 10% of effect sizes, k = 344). Using this reduced dataset, we reran the meta-analyses and tested for the small study bias and time-lag bias again. Distributions of sampling variance and effect sizes are provided in Supplementary Figure 4.


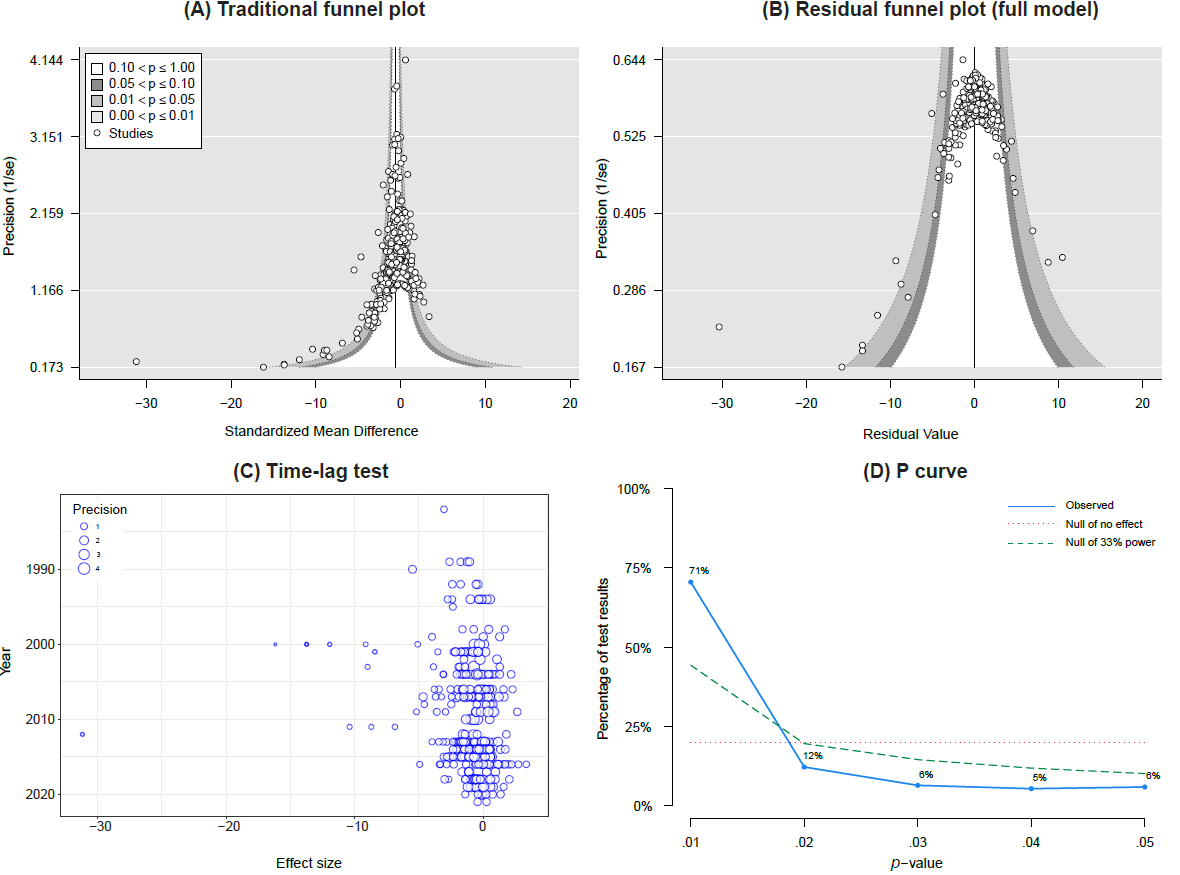


Supplementary Figure 3. Publication bias. (A) Traditional and (B) residual funnel plots show the relationship between effect size (or residual) and precision. (C) Bubble plot shows the publication year associated with effect sizes. Circle sizes represent precision. (D) P-curve plot shows the relationship between *p* values and percentage of test results.


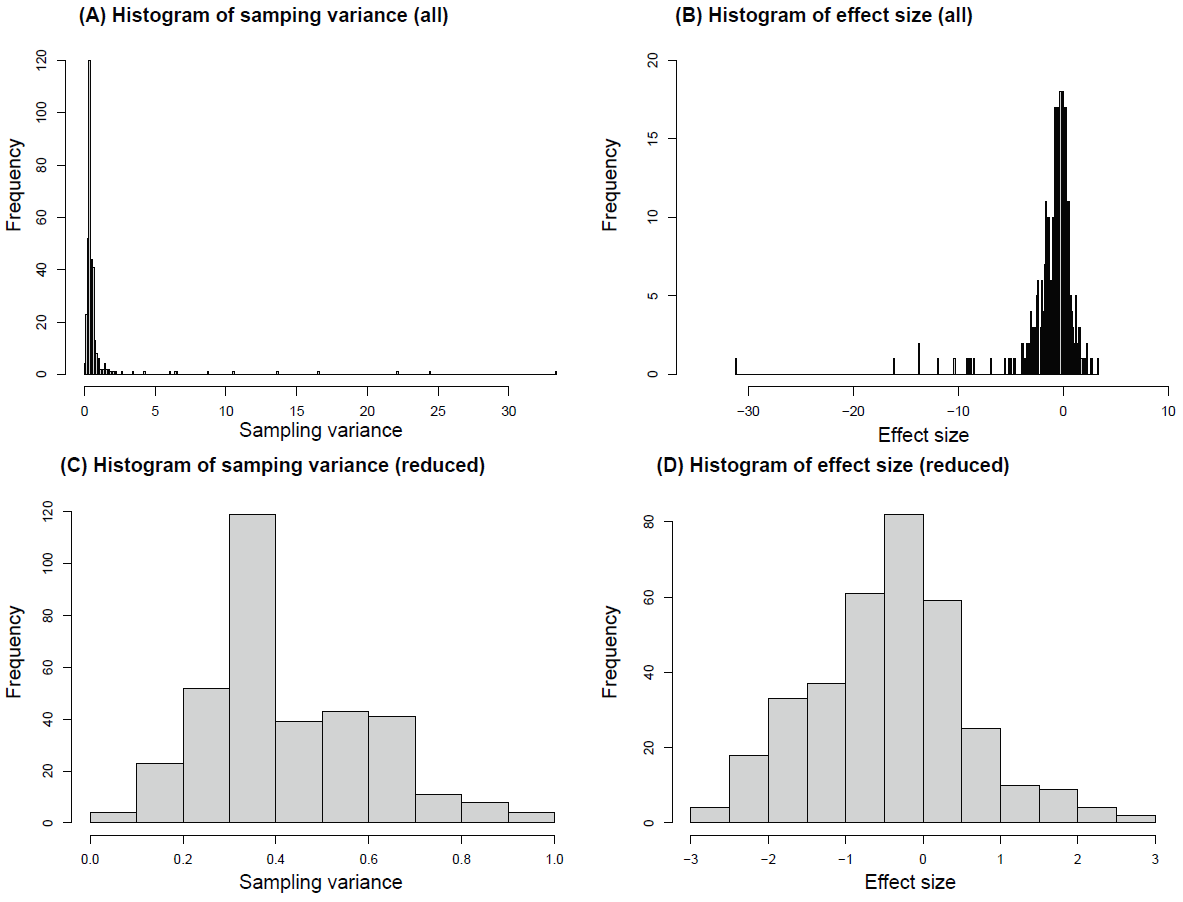


Supplementary Figure 4. Distribution of sampling variance and effect size of full dataset (k = 388) and reduced dataset (excluding studies with top 10% sampling variance or bottom 10% effect sizes, k = 344).

**Supplementary Tables**

Supplementary Table 1. Heterogeneity (%, the amount of variation not attributed to sampling error) with multilevel random effect models for predator foraging behaviour, predator activity, prey activity, and prey antipredator behaviour.

|  | Total | Study | Predator species | Prey species |
| --- | --- | --- | --- | --- |
| Predator foraging behavior | 90.61 | 81.10 | 8.91 | 0.60 |
| Predator activity | 95.43 | 92.79 | 2.00 | 0.64 |
| Prey activity | 24.25 | 6.15 | 12.44 | 5.66 |
| Prey antipredator behaviour | 85.84 | 37.22 | 0.00 | 48.62 |

Supplementary Table 2. Multi-level random effect and mixed-effects models of predator foraging behaviour, predator activity, prey activity, and prey antipredator behaviour. k = number of effect sizes in each group. Estimate = effect size. CI = 95% Confidence interval.

| Response | Moderator |  | Estimate | Lower CI | Upper CI | k |
| --- | --- | --- | --- | --- | --- | --- |
| Predator foraging behaviour | Null |  | -0.74 | -1.41 | -0.07 | 94 |
|  | Number of prey species | Multiple | 0.44 | -1.81 | 2.68 | 5 |
|  |  | Single | -0.85 | -1.55 | -0.15 | 89 |
|  | Setup | Field | 0.44 | -1.81 | 2.68 | 5 |
|  |  | Lab | -0.85 | -1.55 | -0.15 | 89 |
|  | Predator hunting mode | Active | -0.96 | -1.97 | 0.05 | 44 |
|  |  | Ambush | -0.15 | -1.31 | 1.02 | 19 |
|  |  | Mixed | -0.70 | -1.68 | 0.28 | 31 |
|  | System | Freshwater | -1.19 | -2.35 | -0.03 | 46 |
|  |  | Marine | -0.71 | -1.94 | 0.52 | 33 |
|  |  | Terrestrial | -0.29 | -1.49 | 0.91 | 15 |
| Predator activity | Null |  | -1.44 | -3.16 | 0.28 | 53 |
|  | Predator hunting mode | Active | -2.10 | -4.63 | 0.42 | 33 |
|  |  | Ambush | -0.37 | -2.98 | 2.25 | 5 |
|  |  | Mixed | -1.04 | -3.60 | 1.51 | 15 |
|  | System | Freshwater | -2.46 | -5.03 | 0.10 | 26 |
|  |  | Marine | -0.50 | -3.31 | 2.31 | 25 |
|  |  | Terrestrial | -0.13 | -6.33 | 6.08 | 2 |
| Prey activity | Null |  | -0.42 | -0.83 | -0.01 | 12 |
|  | Number of predator species | Multiple | -0.05 | -1.46 | 1.36 | 2 |
|  |  | Single | -0.46 | -0.90 | -0.02 | 10 |
|  | Predator hunting mode | Active | -0.14 | -0.74 | 0.46 | 4 |
|  |  | Ambush | -0.40 | -1.11 | 0.31 | 2 |
|  |  | Mixed | -0.87 | -1.48 | -0.27 | 4 |
|  | Setup | Field | -0.05 | -1.46 | 1.36 | 2 |
|  |  | Lab | -0.46 | -0.90 | -0.02 | 10 |
|  | System | Freshwater | -0.59 | -1.08 | -0.10 | 8 |
|  |  | Marine | -0.05 | -1.44 | 1.35 | 2 |
|  |  | Terrestrial | -0.10 | -0.96 | 0.77 | 2 |
| Prey antipredator behaviour | Null |  | -0.41 | -1.20 | 0.37 | 25 |
|  | Number of predator species | Multiple | 0.22 | -1.42 | 1.85 | 10 |
|  |  | Single | -0.64 | -1.58 | 0.30 | 15 |
|  | Predator hunting mode | Active | -0.61 | -2.22 | 1.00 | 8 |
|  |  | ambush | -0.26 | -2.06 | 1.54 | 3 |
|  |  | mixed | -1.16 | -2.97 | 0.65 | 4 |
|  | Setup | Field | 0.22 | -1.42 | 1.85 | 10 |
|  |  | Lab | -0.64 | -1.58 | 0.30 | 15 |
|  | System | Freshwater | -0.23 | -1.29 | 0.84 | 12 |
|  |  | Marine | -0.22 | -1.58 | 1.13 | 12 |
|  |  | Terrestrial | -2.05 | -4.54 | 0.43 | 1 |

Supplementary Table 3. Multi-level random effect and mixed-effects models of outcome of predator and prey interaction (only single predator species), controlling for predator phylogeny. k = number of effect sizes in each group. Estimate = effect size. CI = 95% Confidence interval.

| Moderator |  | Estimate | Lower CI | Upper CI | k |
| --- | --- | --- | --- | --- | --- |
| Null |  | -0.97 | -1.45 | -0.48 | 327 |
| Number of prey species | Multiple | -0.92 | -1.79 | -0.06 | 29 |
|  | Single | -0.97 | -1.48 | -0.47 | 298 |
| System | Freshwater | -0.95 | -1.61 | -0.29 | 174 |
|  | Marine | -1.01 | -1.67 | -0.34 | 120 |
|  | Terrestrial | -0.92 | -1.98 | 0.13 | 33 |
| Predator hunting mode | Active | -0.94 | -1.52 | -0.36 | 177 |
|  | Ambush | -1.00 | -1.76 | -0.24 | 84 |
|  | Mixed | -0.73 | -1.71 | 0.25 | 37 |
| Setup | Field | -0.79 | -1.47 | -0.12 | 18 |
|  | Lab | -0.99 | -1.47 | -0.50 | 309 |
| Top predator | Absence | -0.95 | -1.45 | -0.46 | 303 |
|  | Presence | -0.37 | -0.97 | 0.23 | 22 |

Supplementary Table 4. Multi-level random effect and mixed-effect models of outcome of predator and prey interaction using reduced data (removing small studies and studies reporting large effects). k = number of effect sizes in each group. Estimate = effect size. CI = 95% Confidence interval

|  |  | Estimate | Lower CI | Upper CI | k |
| --- | --- | --- | --- | --- | --- |
| Null |  | -0.47 | -0.65 | -0.29 | 344 |
| Top predator | Absence | -0.49 | -0.68 | -0.30 | 319 |
|  | Presence | -0.15 | -0.52 | 0.22 | 23 |
| System | Freshwater | -0.51 | -0.81 | -0.22 | 176 |
|  | Marine | -0.41 | -0.68 | -0.15 | 136 |
|  | Terrestrial | -0.56 | -1.09 | -0.02 | 32 |
| Prey species | Multiple | -0.59 | -1.07 | -0.12 | 36 |
|  | Single | -0.45 | -0.65 | -0.25 | 308 |
| Predator species | Multiple | -0.41 | -0.76 | -0.06 | 34 |
|  | Single | -0.48 | -0.67 | -0.29 | 310 |
| Predator mode | Active | -0.45 | -0.68 | -0.22 | 164 |
|  | Ambush | -0.48 | -0.79 | -0.18 | 95 |
|  | Mixed | -0.39 | -0.79 | 0.01 | 35 |
|  | Multiple | -0.40 | -0.97 | 0.17 | 10 |
| Setup | Field | -0.34 | -0.66 | -0.02 | 41 |
|  | Lab | -0.50 | -0.69 | -0.30 | 303 |

Supplementary Table 5. Publication bias analyses of outcome of predator and prey interaction using reduced data (removing small studies and studies reporting large effects).

| Raw Egger’s regression (linear regression) |  | Estimate | SE | t value | Pr(>\|t\|) |
| --- | --- | --- | --- | --- | --- |
|  | Intercept | -0.78 | 0.21 | -3.77 | <0.001 |
|  | sqrt(1/vi) | 0.21 | 0.12 | 1.77 | 0.08 |
|  |  |  |  |  |  |
| Residual Egger’s regression (linear regression) |  | Estimate | Std. Error | t value | Pr(>\|t\|) |
|  | Intercept | -0.30 | 0.22 | -1.33 | 0.18 |
|  | sqrt(1/vi) | 0.22 | 0.13 | 1.70 | 0.09 |
| Time-lag (multilevel mixed-effect model) |  | Estimate | Lower CI | Upper CI |  |
|  | Intercept | -28.76 | -74.29 | 16.77 |  |
|  | Year | 0.01 | -0.01 | 0.04 |  |

Supplementary Table 6. Publication bias analyses of predator foraging behaviour, predator activity, prey activity, and prey anti-predator behaviour.

| Raw Egger’s regression (linear regressions) |  |  | Estimate | SE | t-value | Pr(>\|t\|) |
| --- | --- | --- | --- | --- | --- | --- |
|  | Predator foraging behaviour | Intercept | -5.09 | 0.66 | -7.76 | <0.001 |
|  |  | sqrt(1/vi) | 1.95 | 0.33 | 5.86 | <0.001 |
|  | Predator activity | Intercept | -11.68 | 1.33 | -8.80 | <0.001 |
|  |  | sqrt(1/vi) | 6.06 | 0.86 | 7.04 | <0.001 |
|  | Prey activity | Intercept | 3.53 | 1.24 | 2.85 | 0.02 |
|  |  | sqrt(1/vi) | -2.04 | 0.67 | -3.04 | 0.01 |
|  | Prey anti-predator behaviour | Intercept | -0.17 | 0.42 | -0.41 | 0.68 |
|  |  | sqrt(1/vi) | -0.07 | 0.18 | -0.39 | 0.70 |
| Residual Egger’s regression (linear regression) | Predator foraging behaviour | Intercept | -3.85 | 0.61 | -6.31 | <0.001 |
|  |  | sqrt(1/vi) | 1.82 | 0.31 | 5.86 | <0.001 |
|  | Predator activity | Intercept | -8.34 | 1.13 | -7.36 | <0.001 |
|  |  | sqrt(1/vi) | 4.93 | 0.74 | 6.71 | <0.001 |
|  | Prey activity | Intercept | 1.18 | 0.82 | 1.44 | 0.19 |
|  |  | sqrt(1/vi) | -0.61 | 0.42 | -1.46 | 0.18 |
|  | Prey anti-predator behaviour | Intercept | 0.55 | 0.54 | 1.02 | 0.33 |
|  |  | sqrt(1/vi) | -0.18 | 0.21 | -0.90 | 0.39 |
| Time-lag (multilevel mixed-effect models) |  |  | estimate | Lower CI | Upper CI |  |
|  | Predator foraging behaviour | Intercept | 4.41 | -136.28 | 145.10 |  |
|  |  | Year | 0.00 | -0.07 | 0.07 |  |
|  | Predator activity | Intercept | 8.73 | -327.35 | 344.81 |  |
|  |  | Year | -0.01 | -0.17 | 0.16 |  |
|  | Prey activity | Intercept | 164.90 | -105.44 | 435.23 |  |
|  |  | Year | -0.08 | -0.22 | 0.05 |  |
|  | Prey anti-predator behaviour | Intercept | -22.80 | -194.39 | 148.79 |  |
|  |  | Year | 0.01 | -0.07 | 0.10 |  |

**Supplementary References**

1. Loke, L. H. L., Ladle, R. J., Bouma, T. J. & Todd, P. A. Creating complex habitats for restoration and reconciliation. *Ecological Engineering* **77**, 307–313 (2015).

2. Page, M. J. *et al.* PRISMA 2020 explanation and elaboration: updated guidance and exemplars for reporting systematic reviews. *BMJ* n160 (2021) doi:10.1136/bmj.n160.

3. Rohatgi, A. *WebPlotDigitizer*. (2021).

4. Huey, R. B. & Pianka, E. R. Ecological Consequences of Foraging Mode. *Ecology* **62**, 991–999 (1981).

5. L. James, P. & L. Heck, K. The effects of habitat complexity and light intensity on ambush predation within a simulated seagrass habitat. *Journal of Experimental Marine Biology and Ecology* **176**, 187–200 (1994).

6. Michel, M. J. & Adams, M. M. Differential effects of structural complexity on predator foraging behavior. *Behavioral Ecology* **20**, 313–317 (2009).

7. Savino, J. F. & Stein, R. A. Behavioural interactions between fish predators and their prey: effects of plant density. *Animal Behaviour* **37**, 311–321 (1989).

8. R Core Team. *R: A language and environment for statistical computing*. (2021).

9. Viechtbauer, W. Conducting Meta-Analyses in *R* with the **metafor** Package. *J. Stat. Soft.* **36**, (2010).

10. Bonett, D. G. Meta-analytic interval estimation for standardized and unstandardized mean differences. *Psychological Methods* **14**, 225–238 (2009).

11. Nakagawa, S. & Santos, E. S. A. Methodological issues and advances in biological meta-analysis. *Evol Ecol* **26**, 1253–1274 (2012).

12. Michonneau, F., Brown, J. W. & Winter, D. J. rotl: an R package to interact with the Open Tree of Life data. *Methods Ecol Evol* **7**, 1476–1481 (2016).

13. Paradis, E. & Schliep, K. ape 5.0: an environment for modern phylogenetics and evolutionary analyses in R. *Bioinformatics* **35**, 526–528 (2019).

14. Egger, M., Smith, G. D., Schneider, M. & Minder, C. Bias in meta-analysis detected by a simple, graphical test. *BMJ* **315**, 629–634 (1997).

15. Simonsohn, U., Nelson, L. D. & Simmons, J. P. P-curve: A key to the file-drawer. *Journal of Experimental Psychology: General* **143**, 534–547 (2014).

16. Harrer, M., Cuijpers, P., Furukawa, T. & Ebert, D. D. dmetar: Companion R Package For The Guide Doing Meta-Analysis in R. (2019).

17. Koricheva, J., Gurevitch, J. & Mengersen, K. *Handbook of meta-analysis in ecology and evolution.* (Princeton University Press, 2013).
